# MEK5/ERK5 activation regulates colon cancer stem-like cell properties

**DOI:** 10.1038/s41420-019-0150-1

**Published:** 2019-02-11

**Authors:** Diane M. Pereira, Sofia. E. Gomes, Pedro M. Borralho, Cecília M. P. Rodrigues

**Affiliations:** Research Institute for Medicines (iMed.ULisboa), Faculty of Pharmacy, Universidade de Lisboa, Lisbon, Portugal

## Abstract

Colon cancer has been proposed to be sustained by a small subpopulation of stem-like cells with unique properties allowing them to survive conventional therapies and drive tumor recurrence. Identification of targetable signaling pathways contributing to malignant stem-like cell maintenance may therefore translate into new therapeutic strategies to overcome drug resistance. Here we demonstrated that MEK5/ERK5 signaling activation is associated with stem-like malignant phenotypes. Conversely, using a panel of cell line-derived three-dimensional models, we showed that ERK5 inhibition markedly suppresses the molecular and functional features of colon cancer stem-like cells. Particularly, pharmacological inhibition of ERK5 using XMD8-92 reduced the rate of primary and secondary sphere formation, the expression of pluripotency transcription factors SOX2, NANOG, and OCT4, and the proportion of tumor cells with increased ALDH activity. Notably, this was further associated with increased sensitivity to 5-fluorouracil-based chemotherapy. Mechanistically, ERK5 inhibition resulted in decreased *IL-8* expression and NF-κB transcriptional activity, suggesting a possible ERK5/NF-κB/IL-8 signaling axis regulating stem-like cell malignancy. Taken together, our results provide proof of principle that ERK5-targeted inhibition may be a promising therapeutic approach to eliminate drug-resistant cancer stem-like cells and improve colon cancer treatment.

## Introduction

The identification of stem-like cells within tumors has reshaped our understanding of cancer development, introducing an additional layer of complexity to the concept of intratumoral heterogeneity^1^. The existence of cancer stem cells (CSCs) was demonstrated in several solid tumors, including colon cancer^2–4^. Importantly, CSC populations are characterized by their remarkable potential to perpetuate themselves through self-renewal, while retaining the ability to differentiate into the full repertoire of neoplastic cells forming the heterogeneous tumor mass^5^. Owing to their highly tumorigenic and adaptable phenotype, colon CSCs are currently recognized as the only subset of neoplastic cells holding attributes for tumor initiation, sustained growth, and metastasis formation^6^. Moreover, colon CSCs show increased resistance to conventional antitumor regimens^7–11^, arising as particularly well-suited feeders of tumor regrowth and relapse after initial response to chemotherapy^6^. Adding to the clinical implications of the CSC concept, expression of stemness-associated signatures is associated with worse clinical outcomes in colon cancer patients^12–14^. Elucidation of the molecular players regulating stem-like cell maintenance in colon cancer may therefore translate into new therapeutic strategies to overcome drug resistance and avoid tumor recurrence.

Malignant stem-like cells reproduce many of the signaling programs employed during embryonic development and tissue homeostasis^15^. The extracellular signal-regulated kinase 5 (ERK5 or BMK1) is a non-redundant member of the mitogen-activated protein kinase (MAPK) family that operates within an exclusive MAPK kinase 5 (MEK5)-ERK5 axis to control cell proliferation, survival, differentiation, and motility^16^. Targeted deletion of *Mek5* and *Erk5* in mice provided the first evidence for their essential role in development, leading to embryonic lethality at mid-gestation due to defective endothelial cell function and cardiovascular formation^17–20^. In addition, MEK5/ERK5 signaling has been implicated in the regulation of neurogenic^21–24^, myogenic^25,26^, and hematopoietic^27–29^ differentiation and lineage commitment. Mechanistically, ERK5 was proposed to act independently to maintain naive pluripotency and control cell fate decisions in mouse embryonic stem cells, suggesting multiple critical functions for this kinase during differentiation^30^.

In the intestine, activation of ERK5 is triggered as a bypass route to rescue epithelial cell turnover upon *Erk1/2* ablation^31^; however, the physiological relevance of this cascade in the gastrointestinal tract remains to be elucidated^32^. On the other hand, substantial attention has been given to the link between aberrant MEK5/ERK5 signaling and the pathogenesis of colon cancer^33–36^. Dysregulation of both MEK5 and ERK5 in human tumor samples is associated with more aggressive and metastatic stages of the disease^33–35^, and poorer survival rates^34–36^. Moreover, evidence from different experimental models showed that ERK5-mediated signaling promotes tumor development, metastasis, and chemoresistance^37^, recapitulating the aforementioned features of colon CSCs^6^. However, thus far, no relationship has been established between colon cancer stem-like phenotypes and MEK5/ERK5 signaling.

In the present study, we show that MEK5/ERK5 signaling contributes to sustained stemness in colon cancer, at least in part, through the activation of a downstream NF-κB/IL-8 axis. More importantly, we provide evidence that pharmacological inhibition of ERK5 may be a promising therapeutic approach to eliminate malignant stem-like cells, avoid chemotherapy resistance, and improve colon cancer treatment.

## Results

### MEK5/ERK5 signaling activation correlates with colon cancer stem-like cell phenotypes

Three-dimensional sphere models are widely used to selectively promote the growth of tumor cell populations with stem-like properties^38,39^, representing a functional system for the in vitro discovery of new signaling pathways regulating self-renewal and differentiation in CSCs. In the present study, we used a panel of established human colon cancer cell lines to generate sphere cultures. For this purpose, cells were grown in non-adherent conditions, using serum-free medium supplemented with growth factors. Under this experimental setting, only malignant cells with stem cell features are expected to survive and proliferate, giving rise to free-floating multicellular spheres, also known as tumorspheres^38,39^. After 1 week, HCT116, HT29, SW480, and SW620 cells were shown to efficiently form tumorspheres (Supplementary Figure S1a), which is in agreement with previous observations^40–42^. Additionally, the expression levels of genes involved in intestinal cell differentiation, including *BMP4*, *CDX2*, *AQP3*, and *ADA*, were significantly decreased in tumorsphere cultures, as compared with their adherent counterparts (*p* < 0.05) (Supplementary Figure S1b). On the other hand, the expression profile of the stemness-associated transcripts *SOX2*, *NANOG*, *OCT4*, and *BMI1* was mostly enriched (*p* < 0.05), further confirming that sphere-forming populations were enriched for undifferentiated cells.

**Fig. 1:**
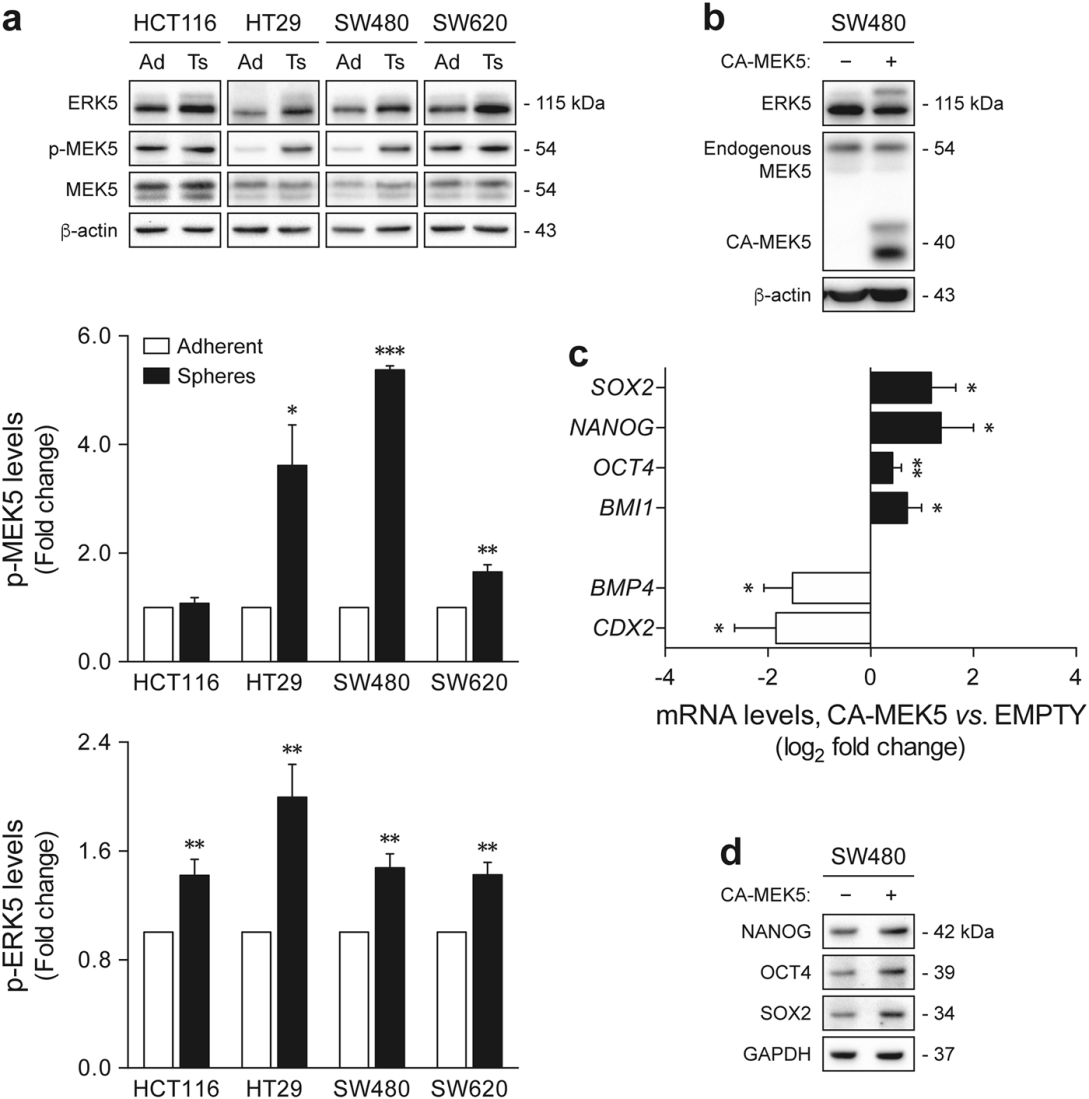
MEK5/ERK5 signaling activation is increased in colon cancer tumorspheres, and MEK5 constitutive activation correlates with a shift toward a stem-like state. **a** HCT116, HT29, SW480, and SW620 cells were cultured under sphere-forming or adherent conditions. MEK5 and ERK5 phosphorylation levels were evaluated by western blot. **b**–**d** SW480 adherent cultures were transfected with CA-MEK5 expression construct or empty vector. **b** MEK5 overexpression and ERK5 activation status were confirmed by immunoblotting. **c** mRNA levels of several stemness- and differentiation-associated markers were determined by qRT-PCR. **d** Steady-state protein levels of NANOG, OCT4, and SOX2 were evaluated by immunoblot analysis. Blots are representative of three independent experiments with similar results. Results are expressed as mean ± standard error of mean from at least three independent experiments. *p < 0.05, **p < 0.01, and ***p < 0.001 from respective adherent cultures (**a**) or empty vector cells (c). Ad Adherent, Ts tumorsphere, CA constitutively active

To determine whether MEK5/ERK5 signaling may be a relevant player in colon cancer stem-like cells, we first analyzed the activation status of these kinases in tumorsphere and matched adherent cultures. Immunoblot analysis showed that, except for HCT116-derived tumorspheres, colon cancer cells grown as spheres had significantly higher levels of MEK5 phosphorylation, compared with monolayer-cultured cells (*p* < 0.05) (Fig. 1a, upper panel). Further, ERK5 phosphorylation was shown to be consistently increased in tumorsphere cultures across all cell lines tested (*p* < 0.01) (Fig. 1a, lower panel), validating that MEK5/ERK5 signaling is overactivated in neoplastic populations enriched for stem-like cells. In turn, forced activation of ERK5 by ectopic expression of a constitutively active mutant of MEK5 (CA-MEK5) in SW480 adherent cultures (Fig. 1b) was associated with lower expression of genes involved in differentiation, and higher levels of stem cell markers, relative to empty vector control cells (*p* < 0.05) (Fig. 1c). Changes in NANOG, OCT4, and SOX2 were confirmed at the protein level (Fig. 1d). Together, these findings demonstrate that MEK5/ERK5 activation correlates with a shift toward an undifferentiated state in colon cancer cells, suggesting that colon cancer stem-like populations may be dependent on ERK5-mediated signaling.

### ERK5 inhibition suppresses colon cancer stem-like cell properties

**Fig. 2:**
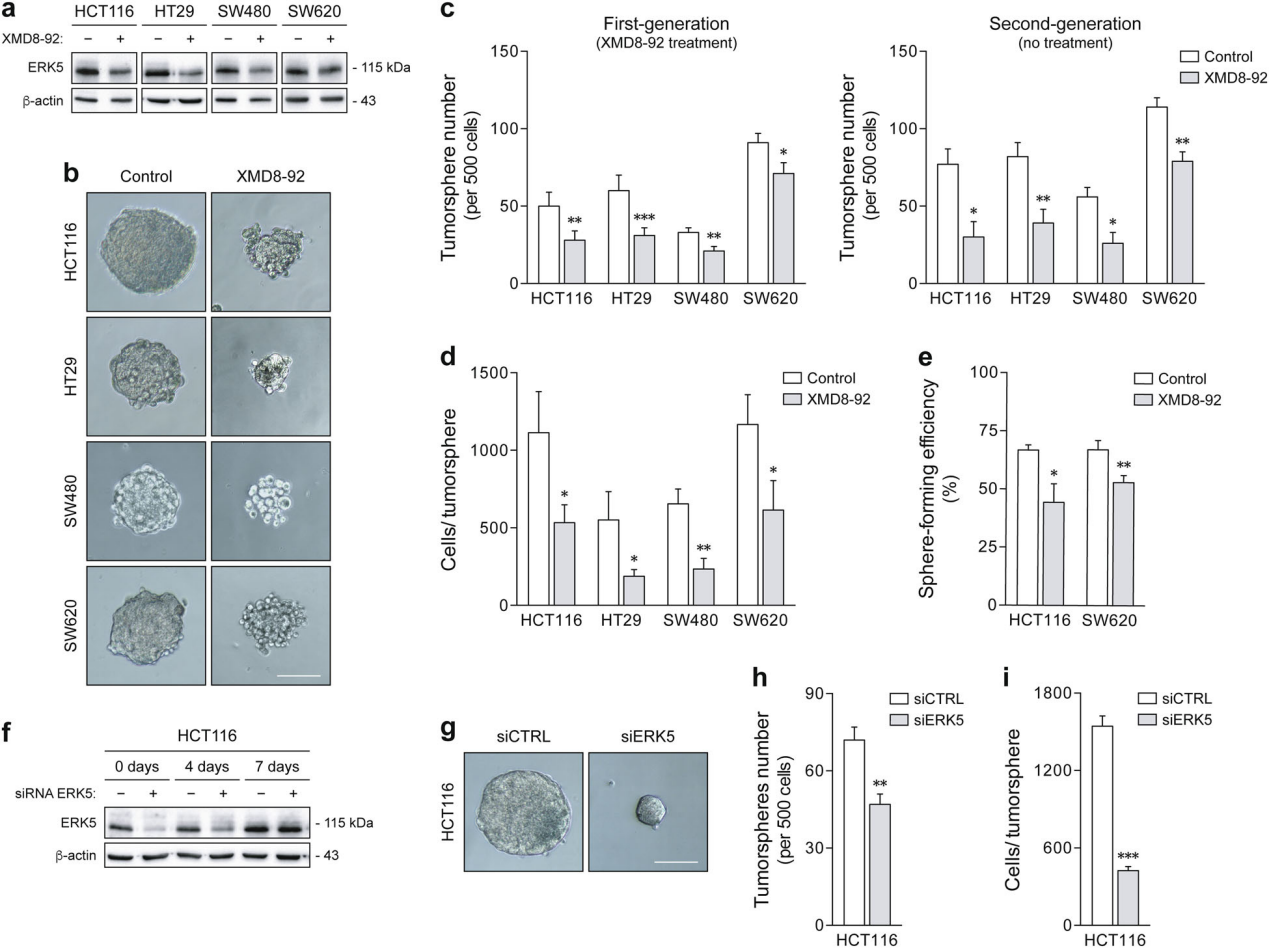
Pharmacological and genetic inhibition of ERK5 impairs sphere formation in colon cancer cells. **a**–**e** HCT116, HT29, SW480, and SW620 cells were cultured for two generations under sphere-forming conditions in the presence or absence of 4 µM XMD8-92. **a** The inhibitory effect of XMD8-92 on ERK5 activation was confirmed by western blot analysis. **b** Representative images of first-generation tumorspheres at ×100 magnification. Scale bar, 100 μm. **c** The rate of tumorsphere formation was measured in primary (with XMD8-92 treatment) and secondary cultures (without additional treatment). **d** Tumorsphere size was determined according to the number of cells per sphere in first-generation cultures. **e** Single-cell assays were performed using HCT116 and SW620 cells to validate the impact of XMD8-92 on clonal sphere formation. **f**–**i** HCT116 cells were transiently transfected with a specific small interfering RNA (siRNA) against ERK5, or control siRNA, and then cultured in non-adherent conditions. **f** ERK5 silencing was monitored by immunoblot analysis during the period of tumorsphere growth. **g** Representative images of 7-day tumorspheres at ×100 magnification. Scale bar, 100 μm. **h** Tumorspheres were scored according to number (left panel) and **i** size (cells per sphere; right panel). Results are expressed as mean ± standard error of mean from at least four independent experiments. *p < 0.05, **p < 0.01, and ***p < 0.001 from vehicle control treatment (**c**–**e**) or control siRNA spheres (**h**, **i**)

To address the functional role of MEK5/ERK5 signaling in colon cancer stem-like cells, HCT116, HT29, SW480, and SW620 cells were plated as tumorspheres, and grown in the presence of XMD8-92, a small-molecule inhibitor of ERK5^43^ (Fig. 2a, b). Self-renewal was then measured according to second-generation sphere formation without any additional treatment. Consistent with our hypothesis, XMD8-92 significantly reduced the frequency of primary and secondary tumorsphere formation (*p* < 0.05) (Fig. 2c). This was further associated with the disruption of sphere morphology and size (*p* < 0.05) (Fig. 2b, d), with minimal effects on cell viability (Supplementary Figure S2), suggesting that besides self-renewal, ERK5 inhibition also impairs the proliferative potential of stem-like malignant cells. Worthy of note, sphere growth was conducted at clonal density (0.25-0.5 cells/μL) to avoid cell aggregation and sphere fusion. Single-cell assays confirmed the clonal origin of tumorspheres^44^, as well as the ability of XMD8-92 to inhibit self-renewal and the rate of sphere formation in both HCT116 and SW620 cells (*p* < 0.05) (Fig. 2e). Finally, to verify the contribution of ERK5 to tumorsphere formation, ERK5 expression was specifically silenced by RNA interference in non-adherent HCT116 cultures (Fig. 2f, g). Interestingly, knockdown of ERK5 led to a marked decrease in the number (*p* < 0.01) and size of HCT116-derived spheres (*p* < 0.001) (Fig. 2h), phenocopying the effects of XMD8-92 treatment. These results demonstrate that ERK5 inhibition depletes the population of sphere-initiating, self-renewing cells in colon cancer cultures.

**Fig. 3:**
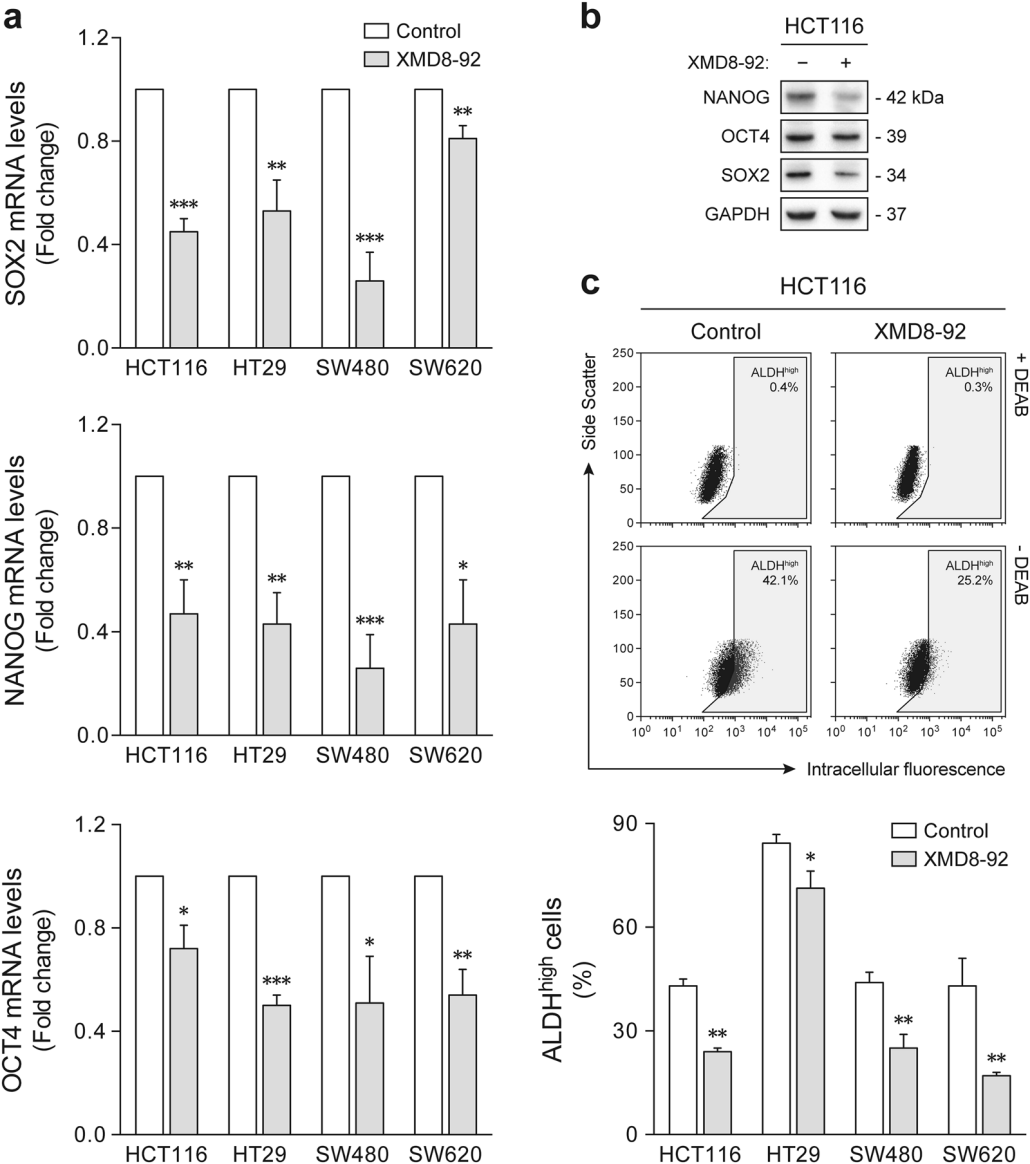
ERK5 pharmacological inhibition suppresses colon cancer stem-like cell molecular features. HCT116, HT29, SW480, and SW620 cells were cultured under sphere-forming conditions in the presence of 4 µM XMD8-92 or dimethyl sulfoxide vehicle control. **a** The mRNA levels of stemness-associated transcription factors were determined by qRT-PCR. **b** Steady-state protein levels of NANOG, OCT4, and SOX2 were confirmed by immunoblot analysis in HCT116-derived tumorspheres. Blots are representative of three independent experiments with similar results. **c** The percentage of cells with high aldehyde dehydrogenase (ALDH) activity was determined by flow cytometry using the Aldefluor assay. Representative side scatter versus green fluorescence intensity plots for HCT116 cells are shown. Results are expressed as mean ± standard error of mean from at least three independent experiments. *p < 0.05, **p < 0.01, and ***p < 0.001 from vehicle control treatment

To investigate the molecular basis underlying the differential frequencies of tumorsphere formation, the expression of core pluripotency transcription factors was next examined. ERK5 inhibition by XMD8-92 resulted in a significant downregulation of *SOX2*, *OCT4*, and *NANOG* in all cellular models under sphere-forming conditions, as assessed by quantitative reverse transcription polymerase chain reaction (RT-PCR) (*p* < 0.05) (Fig. 3a). These results were further confirmed by immunoblot analysis in HCT116-derived tumorspheres (Fig. 3b). Similarly, flow cytometry analysis of aldehyde dehydrogenase (ALDH) activity, a well-characterized marker of colon CSC subpopulations^45^, demonstrated a decrease in the proportion of ALDH-positive cells upon XMD8-92 treatment (*p* < 0.05) (Fig. 3c). Taken together, the aforementioned data demonstrate that ERK5 signaling inhibition suppresses malignant stem-like phenotypes and function, and support the notion that MEK5/ERK5 is required for sustained stemness in colon cancer cells.

### ERK5 pharmacological inhibition sensitizes colon cancer stem-like cells to chemotherapy

**Fig. 4:**
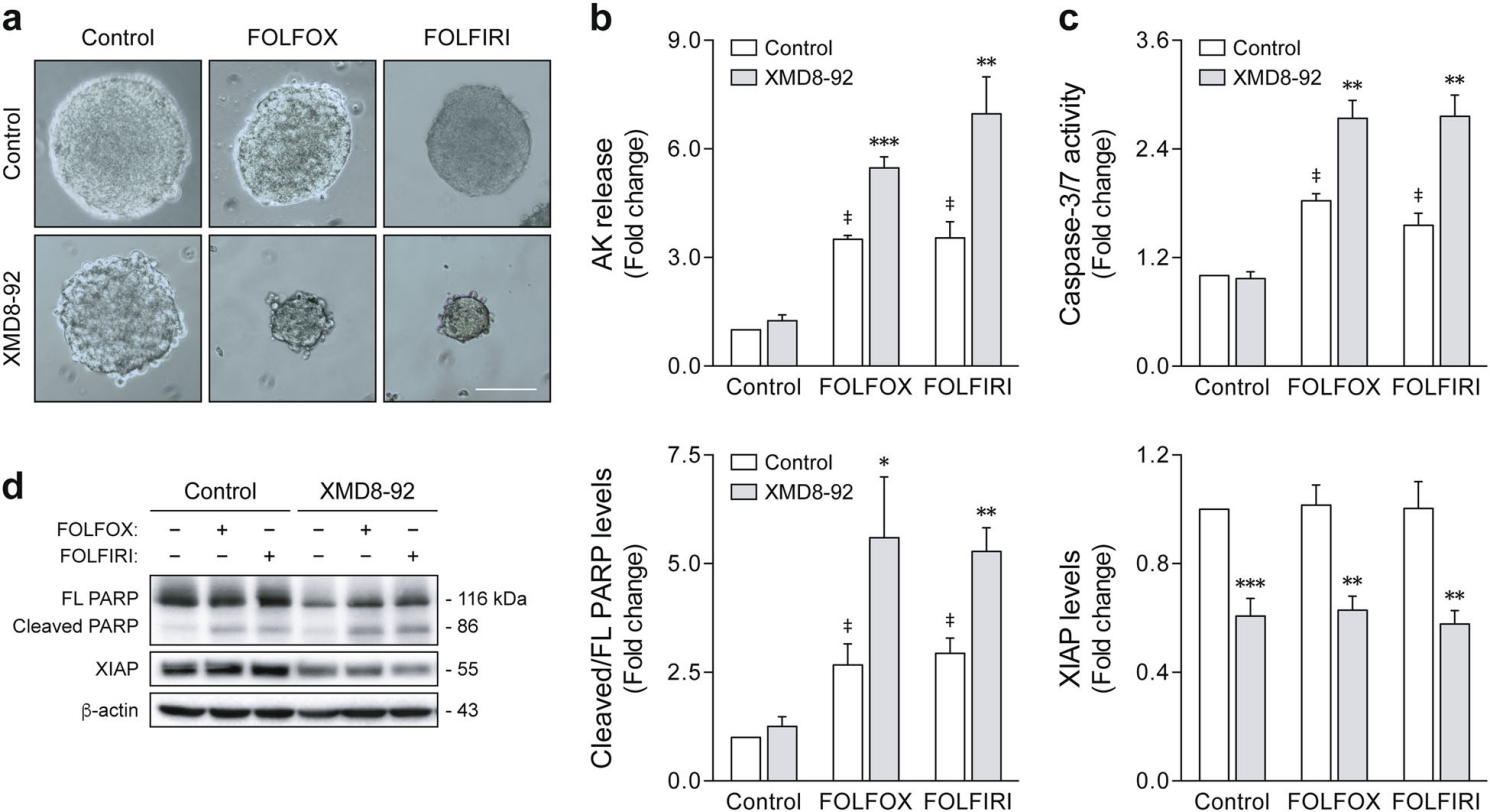
ERK5 pharmacological inhibition sensitizes colon cancer stem-like cells to 5-fluorouracil (5-FU)-based chemotherapy. HCT116 cells were grown for 7 days under sphere-forming conditions, and then treated for 4 days with FOLFOX (50 µM 5-FU plus 1.25 µM oxaliplatin) or FOLFIRI (50 µM 5-FU plus 1 µM irinotecan) alone or in combination with 4 µM XMD8-92. **a** Representative images of chemotherapy-treated tumorspheres at ×100 magnification. Scale bar, 100 μm. **b** Cell death was evaluated according to adenylate kinase (AK) release using the Toxilight assay. **c** Caspase-3/7 activity was measured using the Caspase-Glo 3/7 assay. **d** PARP cleavage and XIAP degradation were evaluated by western blot analysis. Results are expressed as mean ± standard error of mean from at least four independent experiments. ^‡^*p* < 0.01 from vehicle control treatment; *p < 0.05, **p < 0.01, and ***p < 0.001 from FOLFOX/FOLFIRI-single treatment

Colon CSCs have been demonstrated to be highly resistant to standard-of-care chemotherapy^7,8,11^, and combination strategies leading to the suppression of these therapy refractory cells may ultimately translate into improved treatment efficacy and patient outcome. To evaluate the effect of ERK5 inhibition on cancer stem-like cell response to 5-fluorouracil (5-FU)-based chemotherapy, fully formed HCT116-derived tumorspheres were treated with FOLFOX (5-FU plus oxaliplatin) or FOLFIRI (5-FU plus irinotecan), alone or in combination with XMD8-92 (Fig. 4a). Remarkably, XMD8-92-treated tumorspheres showed enhanced sensitivity toward conventional FOLFOX and FOLFIRI treatment, as evidenced by an increase in cell death, compared to chemotherapy alone (*p* < 0.01) (Fig. 4b). Consistent with these observations, the combination of FOLFOX or FOLFIRI with XMD8-92 was further associated with increased caspase-3/7 activity (*p* < 0.01) (Fig. 4c), PARP cleavage (*p* < 0.05) (Fig. 4d, left panel), and XIAP degradation (*p* < 0.01) (Fig. 4d, right panel), demonstrating that ERK5 inhibition primes stem-like malignant populations to chemotherapy-induced apoptosis.

### ERK5 inhibition suppresses interleukin-8 expression through an nuclear factor-kB-dependent mechanism

**Fig. 5:**
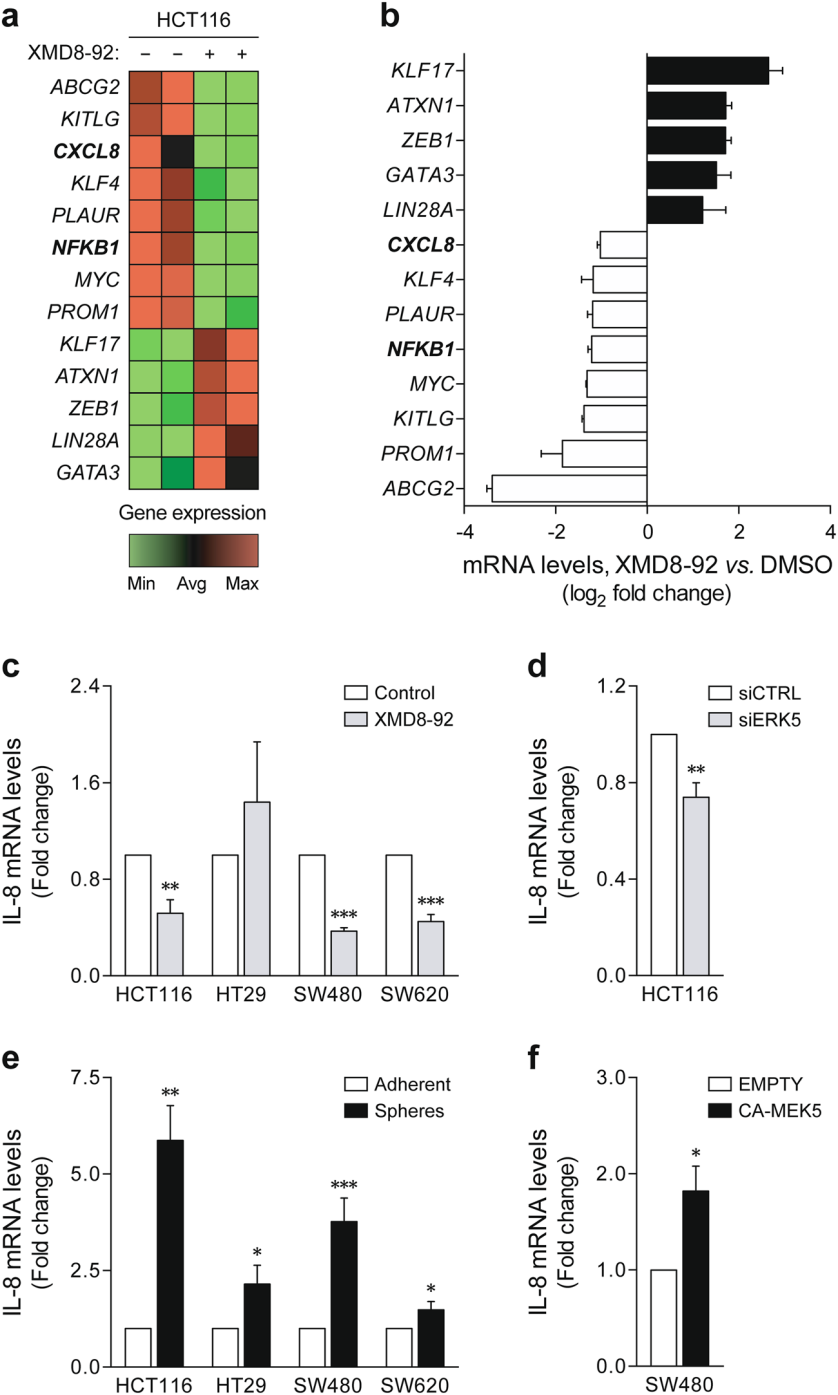
ERK5-mediated signaling regulates *IL-8* expression. **a**, **b** Gene expression profiling was performed in HCT116 tumorspheres using the Human Cancer Stem Cells RT^2^ Profiler PCR Array. **a** Heatmap representation of differentially expressed genes between vehicle- and XMD8-92-treated tumorspheres (green: downregulation; red: upregulation). **b** PCR array results are expressed as mean ± SD log_2_-transformed fold change to vehicle control-treated spheres. **c** The effect of ERK5 inhibition on *IL-8* expression was validated by independent qRT-PCR in HCT116, HT29, SW480, and SW620 tumorspheres treated with XMD8-92 versus vehicle control, and **d** ERK5 small interfering RNA (siRNA) versus control siRNA HCT116 tumorspheres. **e** *IL-8* mRNA levels were further measured in tumorspheres versus adherent cultures, and **f** CA-MEK5-overexpressing versus empty vector SW480 cells. Results are expressed as mean ± standard error of mean from at least three independent experiments. *p < 0.05, **p < 0.01, and ***p < 0.001 from vehicle control treatment (**c**), control siRNA spheres (**d**), parental adherent cultures (**e**), or empty vector cells (**f**). Min minimum, Avg average, Max maximum, CA constitutively active

To identify mechanisms downstream of MEK5/ERK5 that might contribute to stem-like cell maintenance in colon cancer, we performed a comparative PCR array analysis of genes associated with CSC features in HCT116 tumorspheres treated with XMD8-92 or vehicle control. A total of 13 genes were found to be differentially expressed in response to ERK5 inhibition (log_2_-transformed fold change below −1 or above 1) (Fig. 5a, b). In line with the functional and biochemical characterization of tumorspheres, XMD8-92 treatment led to an upregulation of the differentiation factor *GATA3*, and a downregulation of the pluripotency factors *KLF4* and *MYC*, and CSC markers *PROM1*/*CD133* and *PLAUR*/*CD87*. This was further associated with a decrease in the expression of the ATP-binding cassette transporter *ABCG2*. On the other hand, inconsistent effects were found for proliferation and migration-related genes (*KITLG*, *LIN28A*, *KLF17*, and *ZEB1*). Array results were validated by independent quantitative RT-PCR of a selection of differentially expressed transcripts (Supplementary Figure S3).

Apart from the impact of ERK5 inhibition on CSC-associated markers, gene expression profiling also revealed *NFKB1* and the nuclear factor-κB (NF-κB)-regulated *CXCL8/IL-8*^46^ as being downregulated in XMD8-92-treated tumorspheres (Fig. 5a, b). Quantitative RT-PCR confirmed that treatment with XMD8-92 reduced *IL-8* expression in HCT116, as well as SW480 and SW620 tumorspheres (*p* < 0.01) (Fig. 5c). Similar results were observed when genetically silencing ERK5 in HCT116 cells under sphere-forming conditions (*p* < 0.01) (Fig. 5d). These data suggest that elimination of tumor cell populations with stem-like traits through ERK5 inhibition might be a result of downstream *IL-8* repression. Conversely, *IL-8* mRNA levels were enriched in tumorsphere models where MEK5/ERK5 signaling was found to be induced (*p* < 0.05) (Fig. 5e), and in SW480 cells expressing CA-MEK5 (*p* < 0.05) (Fig. 5f), supporting the existence of a functional link between ERK5 activation and interleukin (IL)-8 signaling.

**Fig. 6:**
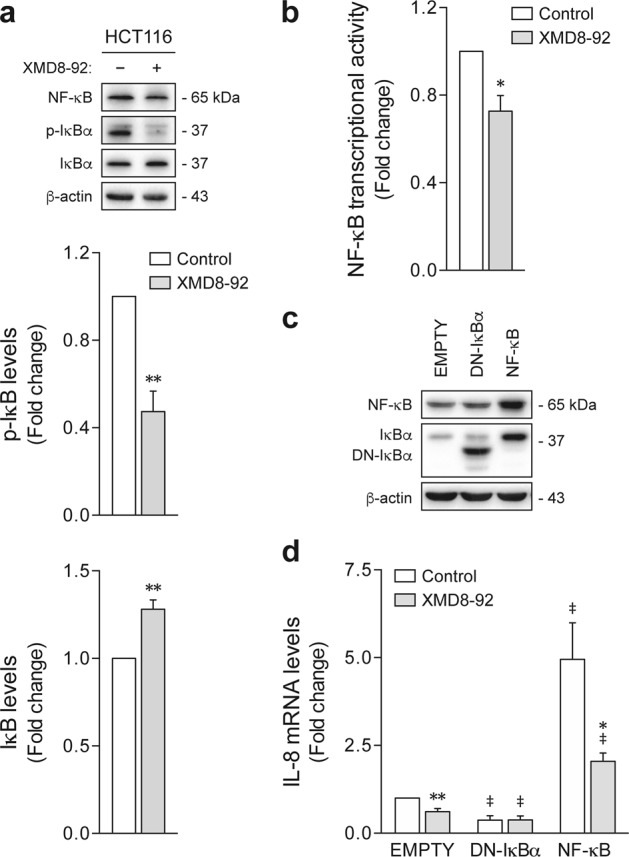
ERK5 pharmacological inhibition blocks NF-kB/IL-8 signaling. **a** HCT116 cells were cultured for 7 days under sphere-forming conditions in the presence of 4 µM XMD8-92 or dimethyl sulfoxide (DMSO) vehicle control. IκB phosphorylation levels were evaluated by western blot. **b** Nuclear factor-κB (NF-κB) transcriptional activity was assayed using an inducible luciferase reporter system. **c**, **d** HCT116 cells were transfected with NF-κB p65 or DN-IκBα expression constructs, and empty vector, and then treated for 48 h with 4 µM XMD8-92. DMSO was used as vehicle control. **c** NF-κB and IκBα overexpression was confirmed by immunoblotting. **d** *IL-8* mRNA levels were determined by qRT-PCR. Results are expressed as mean ± standard error of mean from three independent experiments. *p < 0.05 and **p < 0.01 from vehicle control treatment; ^‡^*p* < 0.01 from empty vector cells. DN dominant negative

Inhibition of ERK5 has been previously shown to suppress IκB phosphorylation, preventing its degradation and subsequent NF-κB activation^33^. Here we investigated the relevance of the interplay between ERK5 and NF-κB signaling pathways in colon CSC. Consistent with previous observations in monolayer-cultured cells^36^, XMD8-92 treatment in HCT116-derived tumorspheres resulted in decreased IκB phosphorylation, and increased IκB protein levels (*p* < 0.01) (Fig. 6a). Moreover, using a luciferase reporter system, NF-κB transcriptional activity was found to be significantly impaired following XMD8-92 exposure (*p* < 0.05) (Fig. 6b), mirroring the repression of *IL-8* upon EKR5 inhibition, and suggesting a possible autocrine ERK5/NF-κB/IL-8 axis driving stem-like cell malignancy. To investigate this further, NF-κB p65 and a dominant-negative-IκBα mutant (DN-IκBα) were respectively used to induce and block NF-κB activity in HCT116 cells (Fig. 6c). Overexpression of NF-κB p65 led to a marked upregulation of *IL-8*, compared to empty vector cells, an outcome that was largely reversed by the addition of XMD8-92 (*p* < 0.05) (Fig. 6d). Conversely, DN-IκBα reduced *IL-8* mRNA levels and abolished the effect of XMD8-92 in the expression of this chemokine. Overall, these data demonstrate that NF-κB is involved in the regulation of IL-8 by ERK5, and provide a functional mechanism by which MEK5/ERK5 signaling contributes to the maintenance of stem-like properties in colon cancer.

## Discussion

In cancers such as those of the colon, tumor initiation and progression occurs through aberrant activation and/or mutation of the same molecular mechanisms that control normal stem cell dynamics^6^, within a network that goes beyond core pluripotency pathways, and is currently recognized to be controlled by multiple protein kinase cascades^47^. Exemplifying this phenomenon is the MEK5/ERK5 signaling pathway, which has been shown to participate in both development and tumorigenesis^16^. In this framework, we hypothesized that ERK5-mediated signaling could contribute to the maintenance of a stem-like population in colon cancer. Particularly, we demonstrate that MEK5/ERK5 activation is increased in several cell line-derived models enriched for malignant stem cells (Fig. 1); and that ERK5 inhibition using XMD8-92 suppresses both self-renewal and the expression of colon CSC-associated markers (Figs. 2 and 3). In line with our results, ERK5 has been previously identified as a critical player for sphere formation and tumor initiation in lung carcinoma cells^48^. Additionally, the suggested role of ERK5 in defining a CSC-like phenotype is consistent with the notion that activation of epithelial-to-mesenchymal transition (EMT) programs induces the acquisition of CSC traits that facilitate metastasis formation^49^. In this regard, we have previously demonstrated that MEK5/ERK5 activation is associated with upregulation of the mesenchymal marker vimentin, promoting colon cancer cell invasive and metastatic behavior in an orthotopic xenograft model^33^. Moreover, several other studies reported that MEK5 and ERK5 regulate EMT features, generation of circulating tumor cells, and metastatic seeding in different tumor contexts^50–52^. Still, further investigation is required to evaluate the possible influence of ERK5-mediated signaling to the molecular mechanisms underlying the connection between EMT and the CSC state.

Apart from the role of malignant stem-like cells in tumor initiation and metastasis, the CSC concept also provides a framework to understand therapy resistance^6^. Evidence from patient-derived three-dimensional cultures and xenograft models indicate that colon CSCs are intrinsically drug-resistant^8–11^. Moreover, the proportion of cells expressing CSC-associated markers was found enriched within residual tumors of colon cancer patients receiving chemoradiotherapy^7^. Therefore, identification of targetable signaling pathways controlling colon cancer stem-like phenotypes will undoubtedly fuel the development of combination regimens to overcome current therapy limitations. We have recently shown that ERK5 inhibition enhances the anticancer properties of 5-FU in a murine xenograft model of colon cancer^36^. Here we extend these earlier studies by revealing that XMD8-92 treatment sensitizes HCT116 cancer stem-like cells to 5-FU-based chemotherapy (Fig. 4), establishing a novel strategy to eliminate drug-resistant populations generated upon phenotypic switching into the CSC state. In parallel, we also found that ERK5 inhibition in tumorspheres leads to downregulation of *ABCG2* (Fig. 5 and Supplementary Figure S3), a drug-efflux pump that is responsible for acquired resistance to both 5-FU^53^ and irinotecan^54^, also contributing to CSC malignant properties^55^. Indeed, and in agreement with our results, the ERK5/MEF2 pathway has been proposed to regulate the expression of several ABC transporters, among which *ABCG2*^56^. It is therefore possible that part of the mechanism behind the increased susceptibility of stem-like colon cancer cells to chemotherapy upon ERK5 inhibition could involve *ABCG2* repression.

Finally, our data demonstrate that NF-κB-mediated *IL-8* expression might be a fundamental element of CSC-like function downstream of MEK5/ERK5 signaling (Figs. 5 and 6). In colon cancer, aberrant expression of the CXC chemokine IL-8, in tumor tissues or in circulation, was shown to be associated with poor differentiation, depth of invasion, and distant metastasis^57–59^. Functionally, IL-8 signaling promotes EMT, stem cell-like traits, and chemoresistance^60–62^. Regarding CSCs, the pro-inflammatory and angiogenic activity of IL-8 is known to be essential for the establishment of a supportive microenvironment for self-renewal and stem-like cell survival^63^. However, according to our experimental conditions, we suggest that IL-8 expression by tumor cells might also contribute to sustained stemness through autocrine signaling. Indeed, a similar feedback loop mechanism has already been proposed for the regulation of colon cancer cell proliferation and migration^60,64^. Strengthening our hypothesis, while NF-κB controls IL-8 expression^46^, this chemokine is in turn responsible for triggering NF-κB transcriptional activity^65^. Consistently, NF-κB signaling has also been linked to CSC-like features in several solid tumors, including glioblastoma, breast, prostate, and non-small cell lung cancer^66–69^. On the other hand, pharmacological inhibition and genetic knockdown of either MEK5 or ERK5 were reported to suppress lipopolysaccharide-, IL-1β-, and tumor necrosis factor-α-induced production of IL-8 in primary human endothelial cells and monocytes^70^. Similarly, we demonstrate that specifically silencing ERK5 recapitulates the effects of XMD8-92-mediated inhibition of ERK5 kinase activity, depleting the population of sphere-initiating cells (Fig. 2), and the expression of *IL-8* in HCT116 tumorspheres (Fig. 5). Nevertheless, we cannot fully exclude that putative XMD8-92 off-target activity may partially account for the observed phenotypic effects of this small-molecule inhibitor against colon cancer stem-like cells^71,72^. Moreover, although cancer cell lines are representative of the different colorectal cancer molecular subtypes, which validates their utility as tools to investigate tumor biology and drug response^73,74^, future studies will be necessary to evaluate the impact of ERK5 inhibition on patient-derived in vitro and in vivo models.

Taken together, our findings provide proof of principle that pharmacological inhibition of ERK5 may be an effective strategy to target self-renewing, drug-resistant colon cancer stem-like cells. Adding to the clinical relevance of this signaling pathway, aberrant MEK5/ERK5 activation also contributes to increased tumor cell proliferation and metastasis^33^, inducing a chemoresistant phenotype in both CSCs and non-CSCs^36^. The plurality of mechanisms through which ERK5 activity drives the process of tumorigenesis reinforces the therapeutic potential of blocking this cascade in colon cancer treatment. Still, heterogeneous tumor masses comprising different populations of differentiated and cancer stem-like cells are expected to be sustained by different oncogenic mechanisms. Therefore, the introduction of ERK5-targeting agents in clinical evaluation should be envisioned as part of combination regimens designed to avoid resistance and tumor recurrence, bringing together conventional cytotoxic drugs and innovative targeted therapies.

## Materials and methods

### Cell culture

Human HCT116, HT29, SW480, and SW620 colorectal carcinoma cell lines were obtained from ECACC (Porton Down, UK), passaged for <6 months after resuscitation, and routinely tested for mycoplasma contamination using Mycoalert detection kit (Lonza, Basel, Switzerland). Cells were cultured in adherent conditions in McCoy’s 5A (HCT116), RPMI 1640 (HT29), or Dulbecco’s modified Eagle’s medium (DMEM) (SW480 and SW620), all supplemented with 10% heat-inactivated fetal bovine serum (FBS) and 1% antibiotic/antimycotic solution (all from Gibco, Thermo Fisher Scientific, Paisley, UK). For the generation of tumorspheres, cells were grown in non-adherent conditions in serum-free DMEM/F12 medium containing 2% B27 supplement, 1% N2 supplement, 1% non-essential amino acids, 1% sodium pyruvate, 1% penicillin-streptavidin, 4 μg/mL heparin, 40 ng/mL recombinant human epidermal growth factor (all from Gibco) and 20 ng/mL recombinant human basic fibroblast growth factor (Peprotech, London, UK). All cell cultures were maintained at 37 °C under a humidified atmosphere of 5% CO_2_.

### Small molecules and chemotherapeutic agents

The ERK5 pharmacological inhibitor XMD8-92 was obtained from Selleckchem (Madrid, Spain) and prepared in dimethyl sulfoxide (DMSO; Sigma-Aldrich, MO, USA). Clinical-grade 5-FU, oxaliplatin, and irinotecan were kindly provided by Hospital São Francisco Xavier (Lisbon, Portugal), and diluted to stock concentrations in phosphate-buffered saline (Gibco, Thermo Fisher Scientific). Stock solutions were aliquoted and stored at −80 and −20 °C, respectively. All subsequent dilutions were freshly prepared in culture medium. Experiments were performed in parallel with DMSO vehicle control. Final DMSO concentration was always 0.1%.

### Cell transfection

For overexpression experiments, CA-MEK5 plasmid (pWPI-MEK5DD; S313D/T317D) was kindly provided by Dr. Robert C. Doebele (University of Colorado, CO, USA)^75^. Constructs for NF-κB p65 (pCMV4 p65)^76^ and DN-IκBα (pCMX IkB alpha M; S32A/S36A)^77^ were obtained from Addgene (#21966 and #12329, respectively). For small interfering RNA (siRNA)-mediated knockdown of ERK5, the MAPK7 Silencer Select was used (#s11149; Applied Biosystems, Thermo Fisher Scientific). HCT116 and SW480 cells were plated at 3 × 10^5^ cells/well on 35 mm dishes and transfected with either 1 μg of plasmid DNA or 80 nM of siRNA using Lipofectamine 3000 (Invitrogen, Thermo Fisher Scientific), according to the manufacturer’s instructions. In both cases, cells were allowed to grow for at least 24 h before further treatment or re-plating for tumorsphere formation.

### Sphere-forming assay

To measure tumorsphere formation, colon cancer cells were plated as single cells in 24-well ultra-low attachment plates (Corning, NY, USA) at 250–500 cells/well, and cultured in 1 mL serum-free DMEM/F12 supplemented with growth factors. After 8 days, spheres were collected, dissociated into single cells, and reseeded as above for secondary sphere formation. For each generation, the number of tumorspheres was determined under an inverted microscope. Additionally, the number of cells per tumorsphere was quantified using trypan blue exclusion assay. Alternatively, cells were sorted at a density of 1 cell/well into 96-well ultra-low attachment plates (BD FACS Aria III, BD Biosciences, CA, USA), and allowed to grow in 200 μL tumorsphere medium. The wells without cells were excluded from analysis one day after plating, and a minimum of 90 wells per condition was considered. Sphere-forming efficiency was calculated after 14 days according to the proportion of wells with tumorspheres versus initially seeded wells. In all cases, cells were allowed to adapt for 24 h and then treated with 4 μM XMD8-92 or DMSO vehicle control, except for second-generation spheres, which were grown without further treatment.

### Aldefluor assay

Cells with high ALDH enzymatic activity were identified using the Aldefluor assay (StemCell Technologies, Grenoble, France) according to the manufacturer’s protocol. In brief, 5,000–10,000 single cells were seeded in 5 mL tumorsphere medium using non-tissue culture-treated 55 mm dishes (Gosselin, Hazebrouck, France), cultured for 24 h, and then treated with either 4 μM XMD8-92 or DMSO vehicle control. Eight-day tumorspheres were collected, dissociated into single cells, resuspended in assay buffer containing 1.5 μM BODIPY-aminoacetaldehyde, and incubated for 40 min at 37 °C. Diethylaminobenzaldehyde (15 μM), a specific ALDH inhibitor, was used as a negative control for each reaction. Samples were then centrifuged, resuspended in fresh assay buffer, and stored on ice until flow cytometric analysis. Sample acquisition was performed in a BD LSRFortessa Cell Analyzer cytometer (BD Biosciences). A total of 10,000 cells were analyzed for each test and control sample pair, and the percentage of ALDH^high^ cells was determined using FlowJo software (version 10.0.7; Tree Star, CA, USA).

### Cell death and caspase activity assays

HCT116 cells were plated in 24-well ultra-low attachment plates and stem cell medium at a density of 500 cells/well. Resulting 8-day tumorsphere cultures were treated for 3 days with FOLFOX (1.25 μM oxaliplatin plus 50 μM 5-FU), or FOLFIRI (1 μM irinotecan plus 50 μM 5-FU) chemotherapeutic regimens^78^, alone or in combination with 4 μM XMD8-92. The in vitro cytotoxic effect of chemotherapy was evaluated using ToxiLight bioassay kit (Lonza) to measure the amount of adenylate kinase (AK) released from plasma membrane-damaged cells into tumorsphere supernatants, following the manufacturer’s instructions. Further, the activity of effector caspases-3 and -7 was measured using Caspase-Glo 3/7 Assay (Promega, WI, USA). For this purpose, tumorspheres were collected at 300 × *g* for 7 min, dissociated into single cells, and resuspended in fresh growth medium. Cell suspensions were then mixed with an equal volume of Caspase-Glo 3/7 reagent, and incubated for 30 min at room temperature, protected from light. Resulting luminescence was measured using the GloMax-Multi+ Detection System (Promega). Experimental AK release and caspase-3/-7 activity levels were normalized by the number of cells per well.

### Quantitative RT-PCR

For gene expression analysis, tumorspheres were grown and treated as described for Aldefluor activity evaluation. Additionally, comparative studies were conducted by culturing colon cancer cells as monolayers in traditional medium supplemented with FBS. Total RNA was extracted using Ribozol (VWR International, PA, USA), treated with RNase-free recombinant DNase I (Roche, Mannheim, Germany), and reverse-transcribed to complementary DNA using the NZY First-Strand cDNA Synthesis Kit (NZYTech, Lisbon, Portugal), all according to the manufacturers’ instructions. Quantitative real-time PCR was performed in 5 μL duplicate reactions on a 384-well QuantStudio 7 Flex Real-Time PCR System (Applied Biosystems, Thermo Fisher Scientific), using the SensiFAST SYBR Hi-ROX kit (Bioline, London, UK), following manufacturer’s protocol. Primer sequences are listed in Supplementary Table S1. For each sample, quantification of gene expression was performed using the relative standard curve method and normalized to *ACTB* levels.

### Total protein isolation and immunoblotting

Total protein extraction and immunoblot analysis were performed as previously described^36^. Briefly, 40 μg of total protein extracts were denatured, separated on 8 or 10% sodium dodecyl sulfate polyacrylamide electrophoresis gels, and transferred onto nitrocellulose membranes. Steady-state protein levels were evaluated using primary rabbit antibodies reactive to ERK5 (#3372), OCT4 (#2750; Cell Signaling Technology, MA, USA), SOX2 (#AB5603, Merck Millipore, MA, USA), p-MEK5 (#sc-135702), PARP (#sc-7150), NF-κB (#sc-372), IκBα (#sc-371), or XIAP (#sc-11426; Santa Cruz Biotechnology, CA, USA); or primary mouse antibodies against MEK5 (#sc-135986), NANOG (#sc-134218, Santa Cruz Biotechnology), or p-IκBα (#9246; Cell Signaling Technology). β-actin (#A5541; Sigma-Aldrich) and GAPDH (#sc-32233) were used as loading controls. Following incubation with appropriate horseradish peroxidase-conjugated secondary antibodies (Bio-Rad Laboratories, CA, USA), the proteins of interest were detected by chemiluminescence using SuperSignal reagents (Pierce, Thermo Fisher Scientific), on a ChemiDoc XRS+ imaging system (Bio-Rad). Densitometric analysis was performed using the Image Lab software (version 5.1; Bio-Rad).

### Gene expression profiling

Differential gene expression between DMSO- and XMD8-92-treated tumorspheres was evaluated using the Human Cancer Stem Cells RT^2^ Profiler PCR Array (PAHS-176Z; Qiagen, MD, USA) according to the manufacturer’s instructions. For each condition, pools were obtained by combining equal amounts of total RNA from five different experiments. Complementary DNA synthesis was performed with 800 ng of DNase I-treated RNA (Roche) and the RT^2^ First Strand Kit (Qiagen). Real-time PCR was run on a 384-well QuantStudio 7 Flex Real-Time PCR System (Thermo Fisher Scientific), using the RT^2^ SYBR Green ROX qPCR master mix (Qiagen). Duplicate reactions for all genes, as well as quality controls for genomic DNA contamination, reverse transcription efficiency, and PCR array reproducibility were included. Data analysis was performed using the GeneGlobe online platform (https://www.qiagen.com/geneglobe/). Relative gene expression over control samples was determined as per the comparative cycle threshold (ΔΔCt) method and normalized to the geometric mean of *B2M* and *HPRT* reference genes (ΔCt = Ct^reference^ − Ct^target^; ΔΔCt = ΔCt^XMD8-92^ − ΔCt^DMSO^). A cutoff value of log_2_-fold change (ΔΔCt) ≥ 1 was defined for the selection of differentially expressed transcripts. Genes with Ct values above 34 or standard deviations between technical replicates superior to 0.5 were excluded from analysis. Results for each detectable gene are shown in Supplementary Table S2.

### NF-κB luciferase reporter assay

NF-κB transcriptional activity was measured using the Cignal NF-κB Pathway Reporter Assay Kit (Qiagen), according to the manufacturer’s specifications. Briefly, HCT116 cells were seeded at 2 × 10^4^ cells/well on 96-well plates and transfected with 100 ng of luciferase construct harboring NF-κB response elements using Lipofectamine 3000 (Invitrogen, Thermo Fisher Scientific). Non-inducible and constitutively expressed firefly luciferase constructs were used as negative and positive controls, respectively. A constitutive Renilla luciferase vector was included in all mixes (40:1) to normalize transfection efficiency and monitor cell viability. Sixteen hours post transfection, cells were treated with 4 μM XMD8-92 or DMSO vehicle control. Luciferase activities were assayed 8 h after treatment using the Dual-Luciferase Reporter Assay System (Promega).

### Statistical analysis

All data are expressed as mean ± standard error of the mean from at least three independent experiments. Statistical significances were determined using unpaired two-tailed Student’s *t*-test. Values of *p* < 0.05 were considered statistically significant.

## Supplementary information

Supp. Material

## References

[1] Shackleton M, Quintana E, Fearon ER, Morrison SJ (2009). Heterogeneity in cancer: cancer stem cells versus clonal evolution. Cell.

[2] Ricci-Vitiani L (2007). Identification and expansion of human colon-cancer-initiating cells. Nature.

[3] O’Brien CA, Pollett A, Gallinger S, Dick JE (2007). A human colon cancer cell capable of initiating tumour growth in immunodeficient mice. Nature.

[4] Dalerba P (2007). Phenotypic characterization of human colorectal cancer stem cells. Proc. Natl Acad. Sci. USA.

[5] Clarke MF (2006). Cancer stem cells--perspectives on current status and future directions: AACR Workshop on cancer stem cells. Cancer Res..

[6] Zeuner A, Todaro M, Stassi G, De Maria R (2014). Colorectal cancer stem cells: from the crypt to the clinic. Cell Stem Cell.

[7] Wilson BJ (2011). ABCB5 identifies a therapy-refractory tumor cell population in colorectal cancer patients. Cancer Res..

[8] Todaro M (2007). Colon cancer stem cells dictate tumor growth and resist cell death by production of interleukin-4. Cell Stem Cell.

[9] Dylla SJ (2008). Colorectal cancer stem cells are enriched in xenogeneic tumors following chemotherapy. PLoS ONE.

[10] Hoey T (2009). DLL4 blockade inhibits tumor growth and reduces tumor-initiating cell frequency. Cell Stem Cell.

[11] Colak S (2014). Decreased mitochondrial priming determines chemoresistance of colon cancer stem cells. Cell Death Differ..

[12] Merlos-Suarez A (2011). The intestinal stem cell signature identifies colorectal cancer stem cells and predicts disease relapse. Cell Stem Cell.

[13] de Sousa EMF (2011). Methylation of cancer-stem-cell-associated Wnt target genes predicts poor prognosis in colorectal cancer patients. Cell Stem Cell.

[14] Dalerba P (2016). CDX2 as a prognostic biomarker in stage II and stage III colon cancer. N. Engl. J. Med..

[15] Karamboulas C, Ailles L (2013). Developmental signaling pathways in cancer stem cells of solid tumors. Biochim. Biophys. Acta.

[16] Nithianandarajah-Jones GN, Wilm B, Goldring CE, Muller J, Cross MJ (2012). ERK5: structure, regulation and function. Cell. Signal..

[17] Sohn SJ, Sarvis BK, Cado D, Winoto A (2002). ERK5 MAPK regulates embryonic angiogenesis and acts as a hypoxia-sensitive repressor of vascular endothelial growth factor expression. J. Biol. Chem..

[18] Regan CP (2002). Erk5 null mice display multiple extraembryonic vascular and embryonic cardiovascular defects. Proc. Natl Acad. Sci. USA.

[19] Yan L (2003). Knockout of ERK5 causes multiple defects in placental and embryonic development. BMC Dev. Biol..

[20] Wang X (2005). Targeted deletion of mek5 causes early embryonic death and defects in the extracellular signal-regulated kinase 5/myocyte enhancer factor 2 cell survival pathway. Mol. Cell. Biol..

[21] Liu L (2006). Extracellular signal-regulated kinase (ERK) 5 is necessary and sufficient to specify cortical neuronal fate. Proc. Natl Acad. Sci. USA.

[22] Li T (2013). Targeted deletion of the ERK5 MAP kinase impairs neuronal differentiation, migration, and survival during adult neurogenesis in the olfactory bulb. PLoS ONE.

[23] Wang W (2014). Genetic activation of ERK5 MAP kinase enhances adult neurogenesis and extends hippocampus-dependent long-term memory. J. Neurosci..

[24] Wang W (2015). Inducible activation of ERK5 MAP kinase enhances adult neurogenesis in the olfactory bulb and improves olfactory function. J. Neurosci..

[25] Dinev D (2001). Extracellular signal regulated kinase 5 (ERK5) is required for the differentiation of muscle cells. EMBO Rep..

[26] Sunadome K (2011). ERK5 regulates muscle cell fusion through Klf transcription factors. Dev. Cell.

[27] Sohn SJ, Lewis GM, Winoto A (2008). Non-redundant function of the MEK5-ERK5 pathway in thymocyte apoptosis. EMBO J..

[28] Wang X (2015). The MAPK ERK5, but not ERK1/2, inhibits the progression of monocytic phenotype to the functioning macrophage. Exp. Cell Res..

[29] Giurisato E (2018). Myeloid ERK5 deficiency suppresses tumor growth by blocking protumor macrophage polarization via STAT3 inhibition. Proc. Natl Acad. Sci. USA.

[30] Williams CA (2016). Erk5 is a key regulator of naive-primed transition and embryonic stem cell identity. Cell Rep..

[31] de Jong PR (2016). ERK5 signalling rescues intestinal epithelial turnover and tumour cell proliferation upon ERK1/2 abrogation. Nat. Commun..

[32] Osaki LH, Gama P (2013). MAPKs and signal transduction in the control of gastrointestinal epithelial cell proliferation and differentiation. Int. J. Mol. Sci..

[33] Simoes AE (2015). Aberrant MEK5/ERK5 signalling contributes to human colon cancer progression via NF-kappaB activation. Cell Death Dis..

[34] Hu B (2012). Expression of the phosphorylated MEK5 protein is associated with TNM staging of colorectal cancer. BMC Cancer.

[35] Diao D (2016). MEK5 overexpression is associated with the occurrence and development of colorectal cancer. BMC Cancer.

[36] Pereira DM (2016). MEK5/ERK5 signaling inhibition increases colon cancer cell sensitivity to 5-fluorouracil through a p53-dependent mechanism. Oncotarget.

[37] Simoes AE, Rodrigues CM, Borralho PM (2016). The MEK5/ERK5 signalling pathway in cancer: a promising novel therapeutic target. Drug Discov. Today.

[38] Weiswald LB, Bellet D, Dangles-Marie V (2015). Spherical cancer models in tumor biology. Neoplasia.

[39] Bielecka ZF, Maliszewska-Olejniczak K, Safir IJ, Szczylik C, Czarnecka AM (2017). Three-dimensional cell culture model utilization in cancer stem cell research. Biol. Rev. Camb. Philos. Soc..

[40] Kanwar SS, Yu Y, Nautiyal J, Patel BB, Majumdar AP (2010). The Wnt/beta-catenin pathway regulates growth and maintenance of colonospheres. Mol. Cancer.

[41] Bitarte N (2011). MicroRNA-451 is involved in the self-renewal, tumorigenicity, and chemoresistance of colorectal cancer stem cells. Stem Cells.

[42] Prabhu VV (2016). Small-molecule prodigiosin restores p53 tumor suppressor activity in chemoresistant colorectal cancer stem cells via c-Jun-mediated DeltaNp73 inhibition and p73 activation. Cancer Res..

[43] Yang Q (2010). Pharmacological inhibition of BMK1 suppresses tumor growth through promyelocytic leukemia protein. Cancer Cell.

[44] Pastrana E, Silva-Vargas V, Doetsch F (2011). Eyes wide open: a critical review of sphere-formation as an assay for stem cells. Cell Stem Cell.

[45] Huang EH (2009). Aldehyde dehydrogenase 1 is a marker for normal and malignant human colonic stem cells (SC) and tracks SC overpopulation during colon tumorigenesis. Cancer Res..

[46] Mukaida N, Okamoto S, Ishikawa Y, Matsushima K (1994). Molecular mechanism of interleukin-8 gene expression. J. Leukoc. Biol..

[47] Fernandez-Alonso R, Bustos F, Williams CAC, Findlay GM (2017). Protein kinases in pluripotency-beyond the usual suspects. J. Mol. Biol..

[48] Song C (2015). Inhibition of BMK1 pathway suppresses cancer stem cells through BNIP3 and BNIP3L. Oncotarget.

[49] Shibue T, Weinberg RA (2017). EMT, CSCs, and drug resistance: the mechanistic link and clinical implications. Nat. Rev. Clin. Oncol..

[50] Mehta PB (2003). MEK5 overexpression is associated with metastatic prostate cancer, and stimulates proliferation, MMP-9 expression and invasion. Oncogene.

[51] Javaid S (2015). MAPK7 regulates EMT features and modulates the generation of CTCs. Mol. Cancer Res..

[52] Pavan S (2018). A kinome-wide high-content siRNA screen identifies MEK5-ERK5 signaling as critical for breast cancer cell EMT and metastasis. Oncogene.

[53] Yuan J (2009). Role of BCRP as a biomarker for predicting resistance to 5-fluorouracil in breast cancer. Cancer Chemother. Pharmacol..

[54] Tuy HD (2016). ABCG2 expression in colorectal adenocarcinomas may predict resistance to irinotecan. Oncol. Lett..

[55] Fang DD (2010). Expansion of CD133(+) colon cancer cultures retaining stem cell properties to enable cancer stem cell target discovery. Br. J. Cancer.

[56] Belkahla S (2018). Changes in metabolism affect expression of ABC transporters through ERK5 and depending on p53 status. Oncotarget.

[57] Ueda T, Shimada E, Urakawa T (1994). Serum levels of cytokines in patients with colorectal cancer: possible involvement of interleukin-6 and interleukin-8 in hematogenous metastasis. J. Gastroenterol..

[58] Terada H, Urano T, Konno H (2005). Association of interleukin-8 and plasminogen activator system in the progression of colorectal cancer. Eur. Surg. Res..

[59] Cacev T, Radosevic S, Krizanac S, Kapitanovic S (2008). Influence of interleukin-8 and interleukin-10 on sporadic colon cancer development and progression. Carcinogenesis.

[60] Bates RC, DeLeo MJ, Mercurio AM (2004). The epithelial-mesenchymal transition of colon carcinoma involves expression of IL-8 and CXCR-1-mediated chemotaxis. Exp. Cell Res..

[61] Ning Y (2011). Interleukin-8 is associated with proliferation, migration, angiogenesis and chemosensitivity in vitro and in vivo in colon cancer cell line models. Int. J. Cancer.

[62] Hwang WL (2011). SNAIL regulates interleukin-8 expression, stem cell-like activity, and tumorigenicity of human colorectal carcinoma cells. Gastroenterology.

[63] Korkaya H, Liu S, Wicha MS (2011). Regulation of cancer stem cells by cytokine networks: attacking cancer’s inflammatory roots. Clin. Cancer Res..

[64] Brew R (2000). Interleukin-8 as an autocrine growth factor for human colon carcinoma cells in vitro. Cytokine.

[65] Manna SK, Ramesh GT (2005). Interleukin-8 induces nuclear transcription factor-kappaB through a TRAF6-dependent pathway. J. Biol. Chem..

[66] Rajasekhar VK, Studer L, Gerald W, Socci ND, Scher HI (2011). Tumour-initiating stem-like cells in human prostate cancer exhibit increased NF-kappaB signalling. Nat. Commun..

[67] Liu M (2010). The canonical NF-kappaB pathway governs mammary tumorigenesis in transgenic mice and tumor stem cell expansion. Cancer Res..

[68] Garner JM (2013). Constitutive activation of signal transducer and activator of transcription 3 (STAT3) and nuclear factor kappaB signaling in glioblastoma cancer stem cells regulates the Notch pathway. J. Biol. Chem..

[69] Zakaria N, Mohd Yusoff N, Zakaria Z, Widera D, Yahaya BH (2018). Inhibition of NF-kappaB signaling reduces the stemness characteristics of lung cancer stem cells. Front. Oncol..

[70] Wilhelmsen K (2015). Extracellular signal-regulated kinase 5 promotes acute cellular and systemic inflammation. Sci. Signal..

[71] Sureban SM (2014). XMD8-92 inhibits pancreatic tumor xenograft growth via a DCLK1-dependent mechanism. Cancer Lett..

[72] Lin EC (2016). ERK5 kinase activity is dispensable for cellular immune response and proliferation. Proc. Natl Acad. Sci. USA.

[73] Schlicker A (2012). Subtypes of primary colorectal tumors correlate with response to targeted treatment in colorectal cell lines. BMC Med. Genomics.

[74] Mouradov D (2014). Colorectal cancer cell lines are representative models of the main molecular subtypes of primary cancer. Cancer Res..

[75] Doebele RC (2009). A novel interplay between Epac/Rap1 and mitogen-activated protein kinase kinase 5/extracellular signal-regulated kinase 5 (MEK5/ERK5) regulates thrombospondin to control angiogenesis. Blood.

[76] Ballard DW (1992). The 65-kDa subunit of human NF-kappa B functions as a potent transcriptional activator and a target for v-Rel-mediated repression. Proc. Natl Acad. Sci. USA.

[77] Van Antwerp DJ, Martin SJ, Kafri T, Green DR, Verma IM (1996). Suppression of TNF-alpha-induced apoptosis by NF-kappaB. Science.

[78] Wielenga MCB (2015). ER-stress-induced differentiation sensitizes colon cancer stem cells to chemotherapy. Cell Rep..

