# Supplementary material for: MEK5/ERK5 activation regulates colon cancer stem-like cell properties
Source: Cell Death Discov. 2019 Feb 11;5:68. doi: 10.1038/s41420-019-0150-1 (PMC6370793; doi:10.1038/s41420-019-0150-1)
Supplement: Supplementary file 1 — Supp. Material [file 41420_2019_150_MOESM1_ESM.pdf]

Supplementary Figure S1

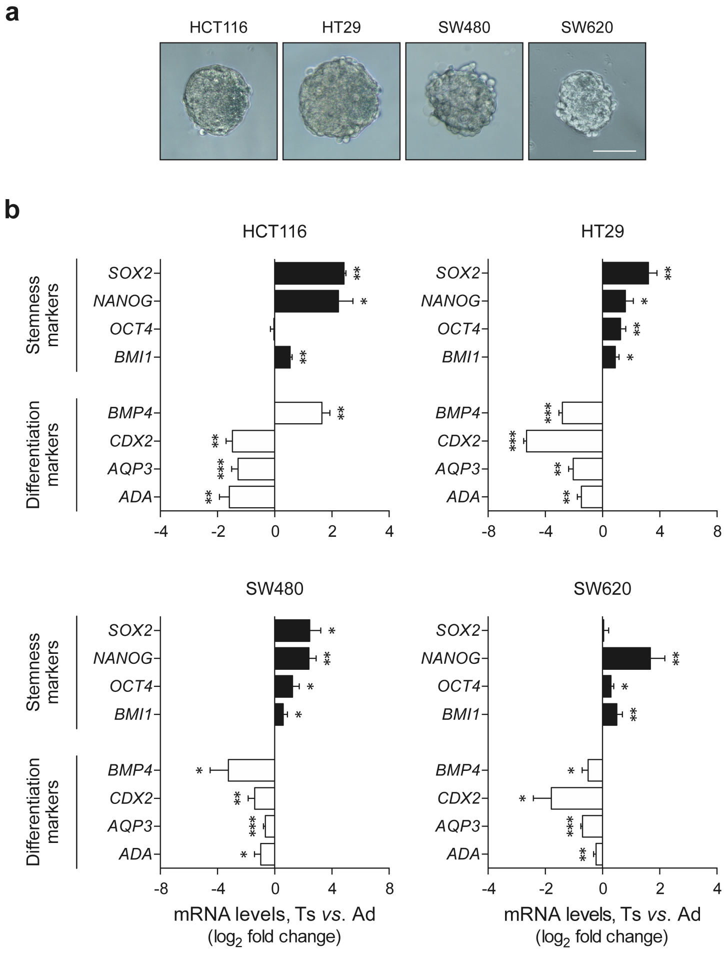

Tumorsphere-forming populations are enriched in stem-like colon cancer cells. HCT116, HT29, SW480 and SW620 cells were cultured under sphere-forming or adherent conditions. (**a**) Representative images of 7-day tumorspheres at 100x magnification. Scale bar, 100 μm. (**b**) The mRNA levels of stemness- and differentiation-associated markers was determined by qRT-PCR. Results are expressed as mean ± SEM $\log_2$-transformed fold change to respective parental adherent cultures, from at least three independent experiments. $^{*}p < 0.05$, $^{**}p < 0.01$ and $^{***}p < 0.001$. Ad, Adherent; Ts, Tumorsphere.

Supplementary Figure S2

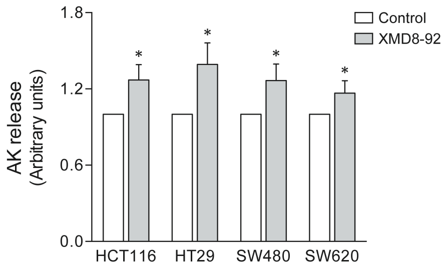

ERK5 pharmacological inhibition has minimal cytotoxic effects in tumorsphere cultures. HCT116, HT29, SW480 and SW620 cells were cultured under sphere-forming conditions in the presence of 4 μM XMD8-92 or vehicle control. General cell death was evaluated according to adenylate kinase (AK) release using the Toxilight assay. Results are expressed as mean ± SEM from four independent experiments. $^{*}p < 0.05$ from vehicle control treatment.

Supplementary Figure S3

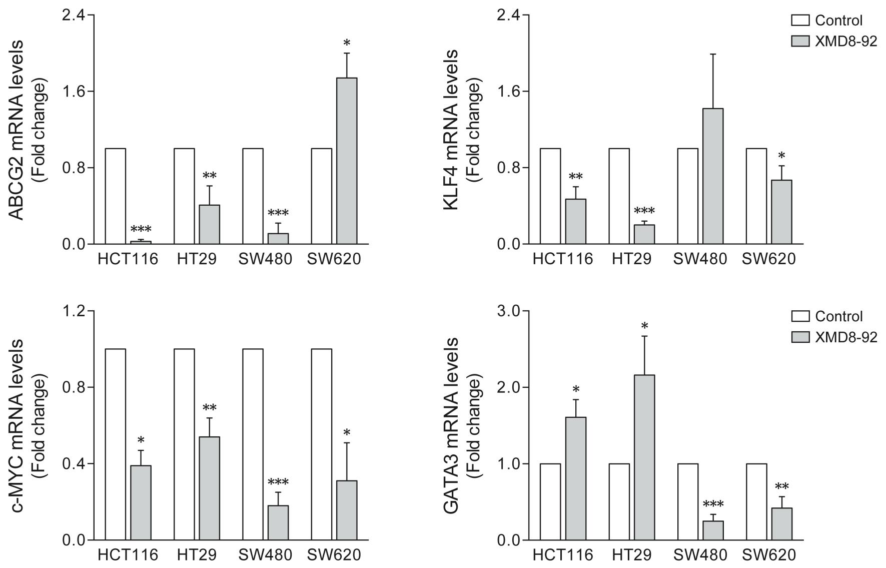

ERK5 pharmacological inhibition modulates the expression of cancer stem cell-associated genes. Quantitative RT-PCR analysis of a selection of differentially expressed genes between vehicle- and XMD8-92-treated HCT116 tumorspheres, as determined according to the Human Cancer Stem Cells RT^2 Profiler PCR Array. Results are expressed as mean ± SEM from at least three independent experiments. $^{*}p < 0.05$, $^{**}p < 0.01$ and $^{***}p < 0.01$ from vehicle control treatment.

Supplementary Table S1. Primer sequences for quantitative RT-PCR

Gene (human)	Forward primer (5'→3')	Reverse primer (5'→3')
ABCG2	ACGAACGGATTAACAGGGTCA	CTCCAGACACACCACGGAT
ACTB	CTGGAACGGTGAAGGTGACA	AAGGGACTTCCTGTAACAACGCA
ADA	GCCTTCGACAAGCCCAAAGTA	CTCTGCTGTGTTAGCTGGGAG
AQP3	CCGTGACCTTTGCCATGTG	CGAAGTGCCAGATTGCATCATAA
BMI1	TCATCCTTCTGCTGATGCTG	CCGATCCAATCTGTTCTGGT
BMP4	GATCCACAGCACTGGTCTTG	GGGATGCTGCTGAGGTTAAA
CDX2	ACTACAGTCGCTACATCACCA	GAAGACACCGGACTCAAGGG
GATA3	TTAACATCGACGGTCAAGGC	GGTAGGGATCCATGAAGCAG
IL8	CTTGGCAGCCTTCCTGATTT	TTCTTTAGCACTCCTTGGCAAAA
KLF4	CCAATTACCCATCCTTCCTG	CGATCGTCTTCCCCTCTTTG
MYC	ACTCTGAGGAGGAACAAGAA	TGGAGACGTGGCACCTCTT
NANOG	TCTGGACACTGGCTGAATCCT	CGCTGATTAGGCTCCAACCAT
OCT4	TCGAGAACCGAGTGAGAGG	GAACCACACTCGGACCACA
SOX2	GCTAGTCTCCAAGCGACGAA	GCAAGAAGCCTCTCCTTGAA

Supplementary Table S2. Analysis of cancer stem cell-related genes using the RT^2 Profiler PCR Array

Gene	XMD8-92 *vs.* Control (log_2 fold change)	Gene	XMD8-92 *vs.* Control (log_2 fold change)
ABCG2	-3,39	*ITGB1*	0,67
ALCAM	0,04	*JAG1*	-0,36
ATM	0,30	*JAK2*	0,93
ATXN1	1,72	*KITLG*	-1,38
AXL	-0,06	*KLF17*	2,66
BMI1	0,14	*KLF4*	-1,18
BMP7	-0,11	*LATS1*	-0,32
CD44	-0,70	*LIN28A*	1,22
CHEK1	-0,10	*MAML1*	0,35
DKK1	0,01	*MERTK*	-0,53
DNMT1	-0,39	*MYC*	-1,31
EGF	0,98	*NANOG*	0,46
EPCAM	-0,37	*NFKB1*	-1,21
ETFA	0,38	*NOTCH2*	0,19
FGFR2	-0,08	*PLAUR*	-1,19
FLOT2	-0,13	*PROM1*	-1,85
FOXP1	0,11	*SAV1*	-0,13
FZD7	0,15	*SIRT1*	0,44
GATA3	1,52	*SMO*	0,71
GSK3B	0,21	*STAT3*	0,33
HDAC1	-0,12	*TGFBR1*	-0,19
ID1	-0,10	*TWIST1*	-0,34
IKBKB	-0,10	*WEE1*	-0,42
CXCL8	-1,03	*WWC1*	-0,66
ITGA2	0,54	*YAP1*	-0,23
ITGA6	0,52	*ZEB1*	1,71
